# Orofacial dysfunction in persons with congenital or childhood-onset neuromuscular disorders

**DOI:** 10.1177/22143602261452338

**Published:** 2026-05-16

**Authors:** Lisa Bengtsson-Stelzer, Anna-Karin Kroksmark, Christina Persson, Lisa Tuomi, Anne-Berit Ekström

**Affiliations:** 1 Department of Health and Rehabilitation, Institute of Neuroscience and Physiology, 70712 The Sahlgrenska Academy, University of Gothenburg, Gothenburg, Sweden; 2 Mun-H-Center, Orofacial Resource Centre for Rare Diseases, Public Dental Service, Västra Götaland Public Dental Service, Gothenburg, Region Västra Götaland, Sweden; 3 Department of Otorhinolaryngology, Head and Neck Surgery, 56749 Sahlgrenska University Hospital, Gothenburg, Region Västra Götaland, Sweden; 4 Department of Paediatrics, Institute of Clinical Science, 70712 The Sahlgrenska Academy, University of Gothenburg, Gothenburg, Sweden; 5 Department of Child Neurology, Queen Silvia Children’s Hospital, 56749 Sahlgrenska University Hospital, Gothenburg, Region Västra Götaland, Sweden; 6 Department of Habilitation & Health, 56749 Sahlgrenska University Hospital, Gothenburg, Region Västra Götaland, Sweden

**Keywords:** neuromuscular disease, dysphagia, feeding, swallowing, mastication, jaw, sialorrhea, dysarthria, speech, facial expression

## Abstract

**Background:**

Some symptoms of orofacial dysfunction, such as dysphagia and dysarthria, are commonly encountered in congenital or childhood-onset neuromuscular diseases (NMD), while others, such as reduced saliva control and difficulties using facial expressions, are not as well researched. Overall, there is a lack of knowledge regarding the extents to which different orofacial functions are affected in persons with NMD.

**Objective:**

To identify orofacial dysfunction profiles at group level for congenital or childhood-onset NMD.

**Methods:**

This database study included 206 individuals in the age range of 3–48 years with 21 different congenital or childhood-onset NMD, categorized as anterior horn cell diseases, neuropathies and myopathies. Orofacial dysfunction profiles from the Nordic Orofacial Test - Screening were extracted from a national database of oral health and orofacial functions in rare health conditions.

**Results:**

In the study population, 70% presented with signs of orofacial dysfunction. Neuropathies had the lowest rate of orofacial dysfunctions (25%), followed by myopathies (72%). Anterior horn cell diseases had the highest rate of orofacial dysfunctions (82%). In all the groups, there were individuals without orofacial dysfunction, except for the subgroups of Spinal Muscular Atrophy type 1 and Duchenne Muscular Dystrophy with age range of 19–49 years in which all the participants (100%) demonstrated orofacial dysfunction according to NOT-S.

**Conclusions:**

This study identifies a considerable proportion of individuals with congenital and childhood-onset NMD exhibiting signs of orofacial dysfunction, indicating the need for further investigation. It also shows to what extents different orofacial functions are affected in subgroups of NMD.

## Introduction

### Orofacial dysfunction in neuromuscular diseases

Neuromuscular diseases (NMD) are defined as diseases that primarily affect the motor unit, i.e., the anterior horn cells of the spinal cord, peripheral nerves, the nerve-muscle junction or the muscle fibers.^1^ The predominant symptom of NMD is muscle weakness. Some forms of the disease are congenital, while other forms manifest in childhood or during adulthood.^2^ While most NMD are rare, together they comprise a group with an overall prevalence in the range of 1/2500–1/3500 and represent significant causes of mortality and morbidity in both children and adults.^3^

Orofacial dysfunction is an umbrella term covering various functional difficulties related to, for example, reduced muscle strength, range of motion, sensory function and/or anatomy of the mouth and facial area, and the consequences of these problems at the activity level including difficulties with eating (chewing, swallowing and handling boluses of different consistencies), using facial expressions, producing the sounds required for speech, oral cleansing, that is clear your mouth after eating, and salivary control.^4^ These impairments of orofacial function tend to co-occur and can have negative impacts on both oral and general health, with consequences that include malnutrition, aspiration, dehydration, communication difficulties, malocclusions and impaired dental health.^4,5^ Orofacial functions can be affected by impairments of the peripheral motor system and cranial nerves that result primarily in oral sensory-motor impairment, as well as secondarily due to difficulties with breathing or neck weakness.^6–8^ Orofacial dysfunction can have a major negative impact on an individual’s well-being and social participation.^9–12^

Several studies have demonstrated that orofacial dysfunction is common in cases of congenital or childhood-onset NMD,^5,8,12–17^ although some symptoms have been studied more extensively than others. A literature review reported that feeding impairment and dysphagia were studied more intensely than other orofacial dysfunctions such as dysarthria and salivary control, and that myopathies (including muscular dystrophies) were more frequently studied than diseases that affect other parts of the motor unit, such as anterior horn cell diseases, neuropathies and neurotransmission-related diseases.^13,15^ A study from The Netherlands described pooled overall prevalence rates of 31.5% for dysarthria and 47.2% for dysphagia for 295 children (age range, 2.6–18 years) with 14 different NMD, with difficulties noted in almost all the disease cases.^12^ Dysphagia and dysarthria vary in severity and timing for different NMD,^12,18^ and various orofacial dysfunctions tend to co-occur.^4,5,12^ There are also reports that objective orofacial measures, such as decreased tongue strength, appear before an individual with NMD is recognized as having functional difficulties, such as dysphagia.^14^ It is common for children and adults with NMD, their caregivers, and health professionals to be unaware of orofacial symptoms such as dysphagia, i.e., prolonged mealtimes and chewing difficulties, or reduced maximum mouth opening.^17,19,20^ This is because the symptoms often develop gradually. It is also common among children and adults with NMD that their caregivers are unaware of the risks and treatment options for orofacial dysfunctions,^12,18,20^ despite the fact that early detection can help prevent complications, reduce morbidity and improve the quality of life.^20^

There are several tests and methods for examining specific aspects of orofacial dysfunction in NMD.^5,7,9,14,17,21–27^ However, for screening of orofacial function from a broader perspective, there is limited material available. Due to the complexity of orofacial functions, several health professions are involved in assessment and treatment of orofacial dysfunction. In such interdisciplinary collaborations there is a great need to establish a mutual language, and common measures in the evaluation of orofacial function. The lack of common criteria for assessing orofacial function was demonstrated in a study of the Scandinavian tests available, reported at the second Nordic Conference on Orofacial Therapy in Gothenburg, Sweden, in 2002. At this conference, a group of representatives from various professions was performed with the task of developing a standardized, comprehensive assessment tool for identifying signs of orofacial dysfunction, later resulting in the Nordic Orofacial Test-Screening (NOT-S) to fulfill this need.^28^ The screening test was intended for use in various syndromes and diseases, as well as in different age groups when orofacial functional difficulties were suspected, possible to perform in any examination setting without the use of special equipment, and easy to use for different health professions. Individuals with NMD such as Spinal Muscular Atrophy (SMA) and Duchenne Muscular Dystrophy (DMD) were included in the group for which the material was originally tested for validity and reliability.^28^ The screening has been used on both children and adults in studies including individuals with congenital and childhood-onset NMD such as Myotonic Dystrophy type 1 (DM1) and DMD.^29,30^

Although it is widely known that orofacial dysfunction is commonly associated with congenital or childhood-onset NMD, most studies have focused on one or a few specific domains of orofacial dysfunction, such as dysphagia or dysarthria, and some diseases are better studied than others.^13^ Therefore, there is a need for studies that investigate the entire spectrum of orofacial dysfunction profiles and that compare these profiles across different NMD.

### Aim

The aim of this study was to identify and describe signs of orofacial dysfunction of subjects with congenital or childhood-onset NMD and to define the orofacial dysfunction profiles in different groups of individuals with congenital or childhood-onset NMD based on primary disease location, for anterior horn cell diseases, neuropathies, myopathies and subgroups thereof.

## Subjects and methods

### Participants

#### MHC database

The Mun-H-Center (MHC) is a Swedish national center for orofacial dysfunction in rare health conditions. The MHC research database was created in 1996 to collect data on oral health and orofacial functions in rare health conditions.^4^ Individuals with rare health conditions living in Sweden can be registered in the database if they give their informed consent. The registrations are made in various contexts, such as during clinical visits for assessment of known orofacial dysfunction, but registrations are also offered in connection with recurring educations and patient group meetings on rare health conditions. Thereof, the MHC database contains data on individuals both with and without known orofacial difficulties. The database collects information through structured interviews and clinical examinations. Results of the NOT-S^28^ have been recorded in the MHC database since 2013. The data contained in the MHC database is what could be extracted for the present study.

A data extraction was performed for all individuals with NMD registered in the MHC database between March 2013 and February 2023, in those cases where NOT-S was used during the registration. Anamnestic information, such as current medication, was also retrieved from participants or their caregivers at time at the database registration using standardized questionnaires. If the individual had participated in registration on several visits, the most-recent visit was selected. The NOT-S examinations were completed by three different speech-language pathologists (SLP) with 10–20 years of clinical experience of NMD and orofacial dysfunction. These SLPs regularly perform co-training in order to be ensure comparability.

**Figure 1.**
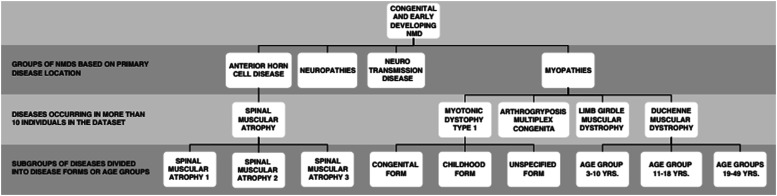
An overview of the classification of groups and subgroups of congenital or childhood-onset neuromuscular diseases used in the present study.

The different NMD are each individually rare health conditions. Therefore, in this study, the NMD were grouped into subcategories. There are several ways to group the NMD. One way is based on where in the motor unit the disease has its primary impact, i.e., anterior horn cell disease, neuropathy, neurotransmission-related disease, and myopathies.^1,5,13^ This categorization has been used in the present study (Figure 1), with subcategories according to an earlier literature review.^13^ The classification of the diseases was accomplished by reaching consensus in a group that comprised a child neurologist, a physiotherapist, and an SLP (author ABE, AKK and LBS), each with more than 10 years of experience working with NMD. In the group of myopathies, muscular dystrophies and arthrogryposis were included. Further classification was based on those diseases for which subgroups were known and that had a prevalence of ten or more participants in the dataset. For DMD, the participants were grouped according to age due to the highly progressive nature of the disease.

Individuals with NMD that do not typically present before the age of 18 years were excluded, including those with Amyotrophic Lateral Sclerosis and the adult form of DM1. Similarly, adult individuals in whom the disease could have debuted after the age of 18 years were excluded, to ensure that the cohort included only cases of congenital or childhood-onset NMD. These criteria resulted in 210 participants in the age range of 3–48 years, with 21 different diseases including subtypes of SMA and DM1. In DM1, individuals were categorized as having the congenital form if symptoms were present at birth, as childhood form if symptoms debuted between the ages of one and ten, and in cases where symptoms appeared before the age of 18 but without further information about when, these were categorized as “unspecified”. In cases where no detailed definition of disease type of congenital myopathy or muscular dystrophy was available in the MHC database, these were classified as “unspecified”. The number of registered individuals with neurotransmission diseases was considered too low (N=3) for presentation at the group level. For one participant with SMA, there was no information on the type of disease. Finally, 206 participants were included in the study (Table 1). Overall, 11 of the 28 participants with SMA and 5 of the of the 69 participants with DMD were receiving ongoing disease-modifying treatments.

**Table 1.** Descriptive statistics for the dataset extracted from the MHC database. Groups of congenital or childhood-onset neuromuscular diseases based on primary disease location, diseases (and, if known, subgroups of disease), numbers of participants, age ranges and comments about disease-modifying treatments.

Congenital or childhood-onset neuromuscular disease		N	Age range, in years	Disease-modifying treatment
**Anterior horn cells disease (N=28)**	Spinal Muscular Atrophy type 1	7	3–13	n=5 treated with Nusinersen
				n=1 treated with Risdiplam
	Spinal Muscular Atrophy type 2	13	5–43	n=5 treated with Nusinersen
				n=1 treated with Risdiplam
	Spinal Muscular Atrophy type 3	8	4–18	n=1 treated with Nusinersen
				n=1 treated with Risdiplam
**Neuropathies (N=12)**	Axonal Motoric Neuropathy	2	21	​
	Charcot-Marie-Tooth	9	4–15	​
	Neuropathy, Congenital Hypomyelination	1	18	​
**Myopathies, including muscular dystrophies and arthrogryposis (N=166)**	Arthrogryposis Multiplex Congenita	15	5–37	​
	Becker Muscular Dystrophy	4	3–14	​
	Bethlem Myopathy	3	8–14	​
	Congenital fiber-type disproportion	1	7	​
	Congenital Muscular Dystrophy	3	8–19	​
	Congenital Myopathy, unspecified	5	6–18	​
	Duchenne Muscular Dystrophy	69	4–48	n=5 treated with Ataluren
	Emery-Dreifuss Muscular Dystrophy	1	18	​
	Facioscapulohumeral muscular dystrophy	3	11–20	​
	Limb-Girdle Muscular Dystrophy	12	7–17	​
	Muscular Dystrophy, unspecified	3	13–19	​
	Myotonic Dystrophy type 1, congenital	13	3-28	​
	Myotonic Dystrophy type 1,childhood-onset	13	10–32	​
	Myotonic Dystrophy type 1, unspecified	14	3–19	​
	Nemaline myopathy	7	5–12	​

#### Nordic orofacial test – screening

The NOT-S is a validated and reliable screening tool for orofacial dysfunction in subjects from 3 years of age.^28,30^ It has been translated into 20 languages and used in international studies to screen for orofacial dysfunctions in children and adults. The NOT-S enables the development of an individual or group-level orofacial dysfunction profile. NOT-S is divided into two parts, with six domains in each. The first part comprises: *NOT-S interview I-VI*, which is patient-reported measurements that include the domains of *I. Sensory function, II. Breathing, III. Habits, IV. Chewing and Swallowing, V. Drooling*, and *VI. Dryness of the Mouth.* The second part is a clinical examination, whereby *NOT-S examination 1-6* includes the domains of *1. Face at Rest, 2. Nose Breathing, 3. Facial Expression, 4. Masticatory Muscles and Jaw Function*, *5. Oral Motor Function*, and *6. Speech*. Each domain includes one to five items. The scoring for each item is based on *“yes”* or *“no”* answers. One or more positive answers in a domain generates a dysfunction score. The more domains of orofacial functions that are negatively affected, the higher the NOT-S total dysfunction score. The maximum NOT-S dysfunction score is 12. When assessing children aged between 3 and 5 years, the speech assessment is adjusted for age. Typically developing children (>3 years) have a mean score of <2.^31^ Based on this, a NOT-S total dysfunction score of ≥2 was chosen as the cut-off value for orofacial dysfunction in this study. NOT-S has proven useful for evaluating the outcomes of habilitation, rehabilitation, and surgical interventions in the orofacial region.^30^ However, the NOT-S neither does not assess the degree of difficulty within each domain, only whether it is impaired or not. NOT-S cannot be used to distinguish different types of motor speech disorders. NOT-S is free of charge and is available for download.^32^ A table summarizing NOT-S is provided in Supplemental materials.

#### Intra- and inter-rater reliability levels

For 46 participants (20%), the NOT-S was video-recorded and evaluated at the time of registration by the first author (LBS). To calculate the intra-rater reliability, LBS performed a second NOT-S assessment. Inter-rater reliability assessments were made by an SLP with over 20 years of experience in NMD. The inter-rater assessments were preceded by a co-assessment of four recordings of individuals with NMD that were not included in the study’s dataset. The intra-rater reliability was calculated to have 87.8% agreement with variation in the range of 84.8%–95.7% for the different domains. The inter-rater reliability was calculated to have 86.6% agreement with variation in the range of 82.2%–100%, with the exception of the *Speech* domain, which achieved 58.7% agreement.

### Ethical considerations

The study was conducted in accordance with the Declaration of Helsinki and was approved by the Swedish Ethical Review Authority (Dn. 544–11 and Dn. 2023-00839-02). Participants gave their informed consent before registration in the MHC database. For participants <18 years of age, a guardian’s signature was required.

## Results

### NOT-S orofacial dysfunction profiles for the total study population

**Figure 2.**
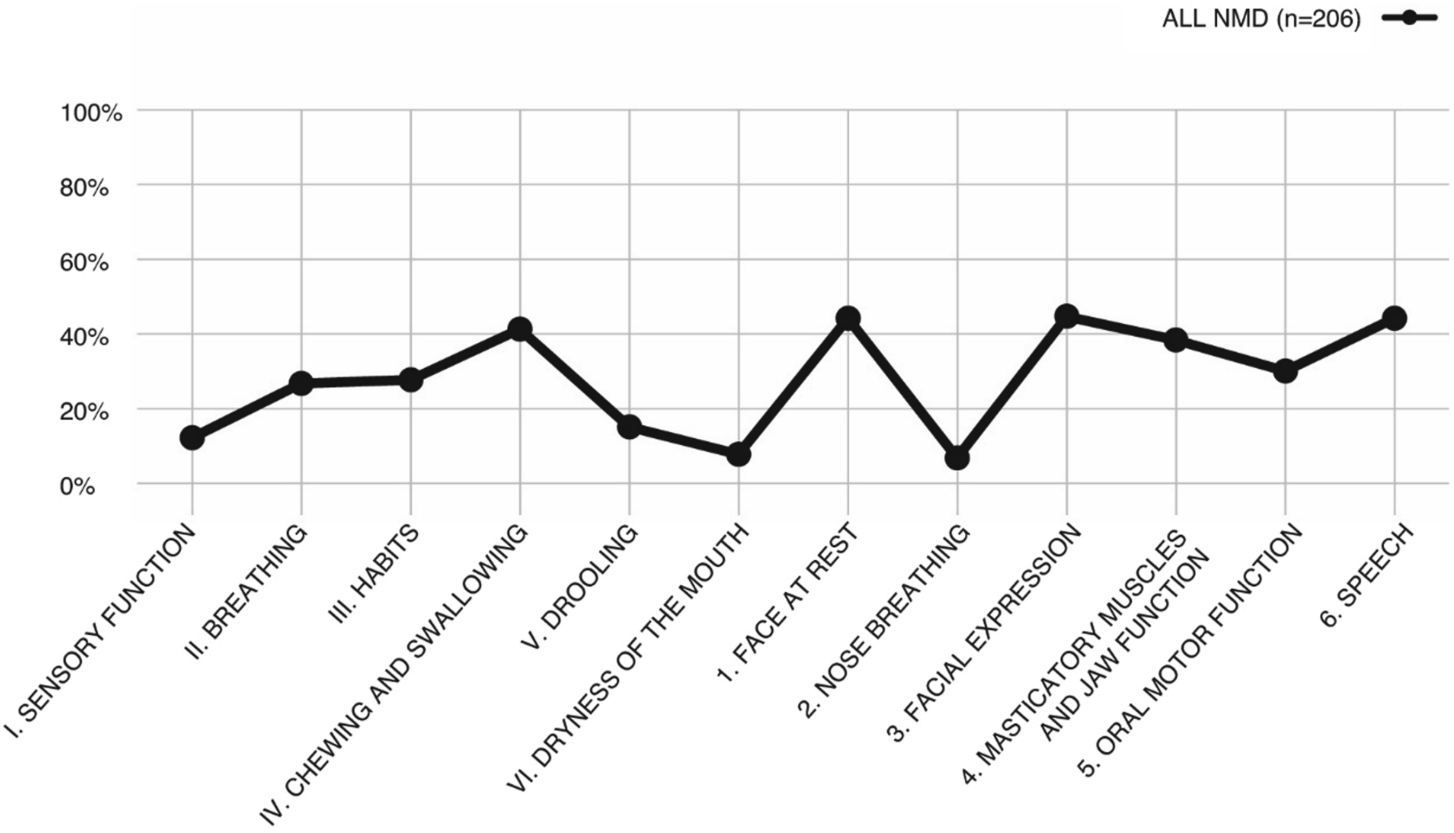
NOT-S orofacial dysfunction profiles with percentages of participants with dysfunction for each domain in the total study population of individuals with congenital or childhood-onset neuromuscular diseases in the MHC database.

Overall, 145/206 (70%) of the total study population presented with scores equivalent to orofacial dysfunction (i.e., NOT-S total dysfunction score of ≥2). Of these, three individuals were under 5 years of age. The median NOT-S total dysfunction score was 3 and the range was 0–10 (Table 2). In 8/12 domains, the NMD group demonstrated orofacial dysfunction at a rate of 25% or higher (Figure 2). The most-common dysfunctions were in the domains of *Facial expression* (45%), *Speech, Face at Rest* (44%), and *Chewing and Swallowing* (41%). A table summarizing the percentage of participants who demonstrated dysfunction in each domain is provided in Supplementary.

**Table 2.** NOT-S total orofacial dysfunction score (median, interquartile range, Min;Max) for the total study population of individuals with congenital and childhood-onset neuromuscular diseases and subgroups thereof.

Congenital or childhood-onset neuromuscular disease	NOT-S Total dysfunction score Median (interquartile range) Min;Max
NMD, total study population (N=206)	3 (1–5) 0;10
^a^ Anterior horn cell disease, All (N=28)	5 (2-7) 0;8
^c^ Spinal Muscular Atrophy type 1 (n=7)	7 (6–8) 3;8
^c^ Spinal Muscular Atrophy type 2 (n=13)	5 (3–6) 1;8
^c^ Spinal Muscular Atrophy type 3 (n=8)	2 (1–5) 0;7
^a^ Neuropathies (N=12)	0 (0-2) 0;7
^a^ Myopathies (N=166)	3 (1-5) 0;10
^b^ Limb-Girdle Muscular Dystrophy (n=12)	1 (0-2) 0;3
^b^ Arthrogryposis Multiplex Congenita (n=15)	2 (1-5) 0;9
^b^ Myotonic Dystrophy type 1 (n=40)	6 (3-7) 0;10
^c^ Congenital form (n=13)	7 (5-7) 0;9
^c^ Childhood form (n=13)	4 (2-6) 1;7
^c^ Unspecified form (n=14)	6 (3-7) 2;10
^b^ Duchenne Muscular Dystrophy (N=69)	3 (1-4) 0;8
^c^ 3–10 years of age (n=28)	3 (1-3) 0;5
^c^ 11–18 years of age (n=33)	2 (1-4) 0;5
^c^ 19–49 years of age (n=8)	4 (3-5) 2;8

^a^Groups of congenital or childhood-onset neuromuscular diseases based on primary disease location.

^b^Myopathies occurring in more than 10 individuals in the dataset (myopathies with fewer than ten individuals in each diagnostic group are not included as a subgroup in the table).

^c^ *Subgrouping of diseases into disease forms or age groups.*

A comparison of the children and adults revealed a high prevalence of orofacial dysfunction in both groups, with almost all the adults showing impairments. In individuals in the age range of 3–18 years (N=176), 66% had ≥2 total dysfunction scores and the most-common difficulties concerned *Facial expression*, *Face at Rest* (44%) and *Speech* (39%). For individuals aged ≥19 years (N=30), 93% presented with orofacial dysfunction. The most frequently occurring domains were: *Speech* (73%); *Masticatory muscles and jaw function* (70%); and *Chewing and swallowing* (57%).

In the group with anterior horn cells disease, 12 individuals had respiratory aids, and 11 individuals were fed entirely or partially with artificial nutrition. In the group with neuropathies, one individual had respiratory aids, and one individual were fed entirely or partially with artificial nutrition. In the group with myopathies 25 individuals had respiratory aids and 19 individuals were fed entirely or partially with artificial nutrition.

### NOT-S orofacial dysfunction profiles of patients with NMD based on primary disease location

**Figure 3.**
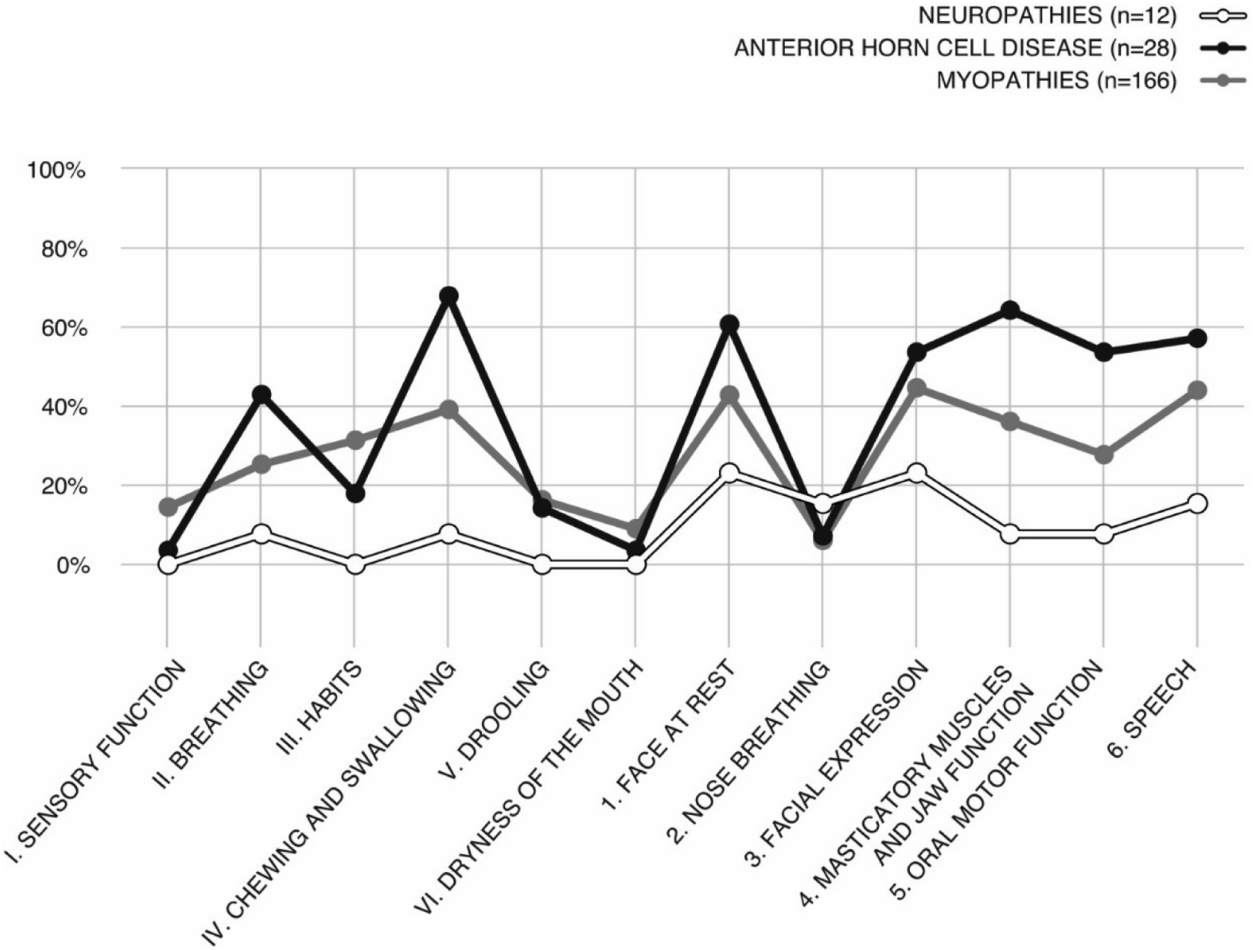
NOT-S orofacial dysfunction profiles with percentages of participants with dysfunction for each domain of the following neuromuscular diseases subtypes: anterior horn cell disease; neuropathies; and myopathies, including muscular dystrophies and arthrogryposis in the MHC database.

In the comparison of NOT-S Total Dysfunction Scores among the three NMD groups defined by primary disease localization, the neuropathy group exhibited the lowest proportion of individuals with signs of orofacial dysfunction (3/12, 25%), followed by the myopathy group (119/166, 72%). Signs of orofacial dysfunction was most commonly observed in anterior horn cell diseases (23/28, 82%). At the individual level, there was, within all three groups, those who neither reported problems in the interview part nor displayed orofacial dysfunction in the examination part of the NOT-S (Table 2, Figure 3).

Among the cases with neuropathies, the most-common dysfunctions occurred in the domains of *Face at Rest* (23%), *Facial Expression* (23%), and *Speech* (15%). Among the subjects with myopathies, the most-common dysfunctions occurred in the domains of *Facial Expression* (45%), *Speech* (44%), and *Face at Rest* (43%). The most-frequent dysfunctions observed in the group with anterior horn cell diseases were in the domains of *Chewing and Swallowing* (68%), *Masticatory Muscles and Jaw Function* (64%), and *Face at Rest* (61%).

### NOT-S orofacial dysfunction profiles of patients with anterior horn cell disease

**Figure 4.**
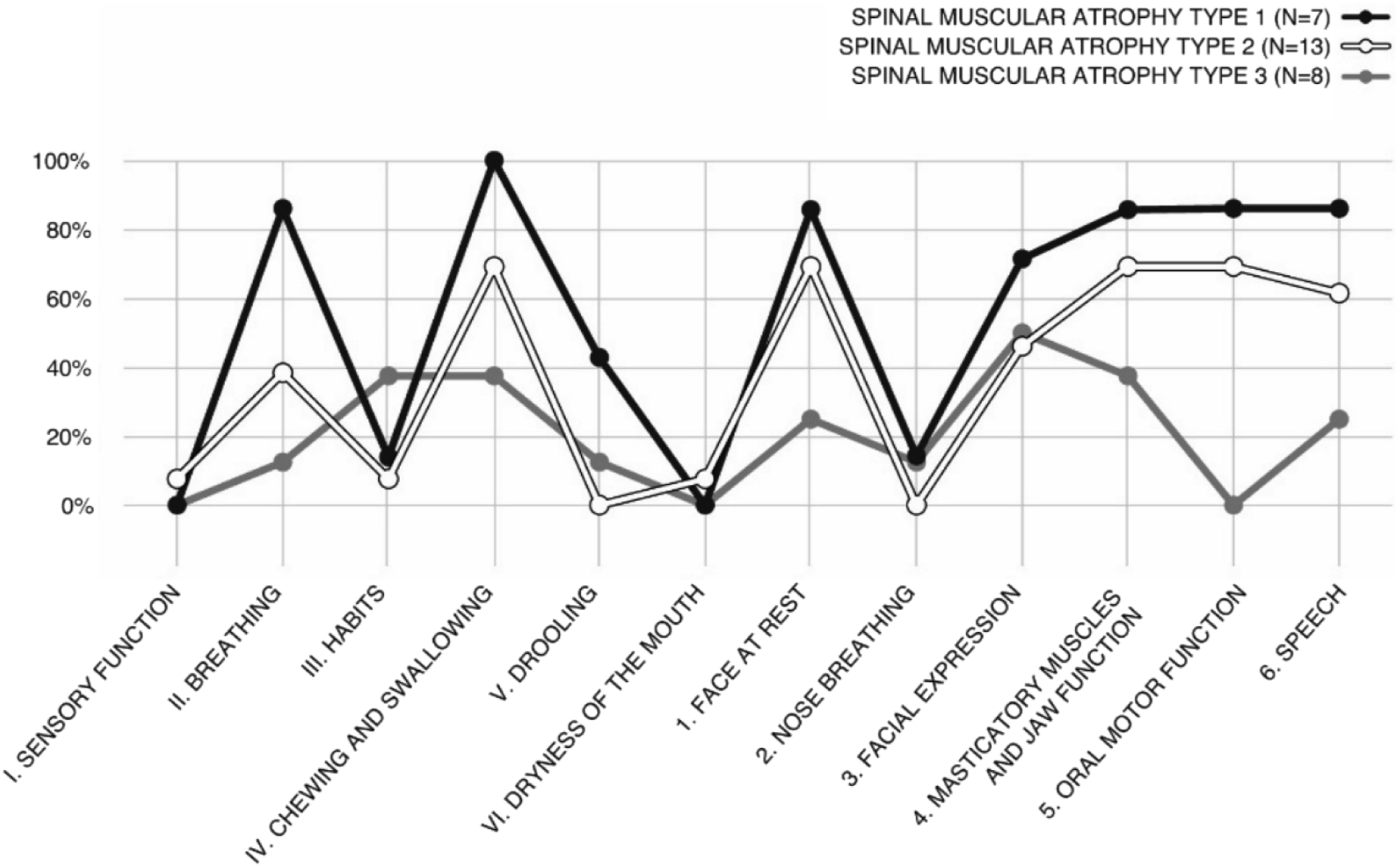
NOT-S orofacial dysfunction profiles with percentages of participants with dysfunction for each domain in the subgroups of anterior horn cell disease (spinal muscular atrophy types 1–3) in the MHC database.

The anterior horn cell disease group contained individuals with SMA type 1-3. Patients with SMA types 1 and 2 showed similar patterns, while the SMA type 3 group demonstrated a slightly different profile. SMA type 1 presented with the most-severe orofacial dysfunctions, where all participants (7/7, 100%) having a total NOT-S score ≥2. For SMA type 2, 12/13 participants (92%) showed signs of orofacial dysfunctions, whereas for SMA type 3, 4/8 participants (50%) had a total score that corresponded to orofacial dysfunction (Figure 4, Table 2).

In SMA type 1, the most-common dysfunctions occurred in the domains of *Chewing and Swallowing* (100%), and *Masticatory Muscles and Jaw Function, Oral Motor Function*, and *Speech, Breathing* (86% for each domain). The most-frequent dysfunctions in the SMA type 2 group were in the domains of *Chewing and Swallowing*, *Face at Rest*, *Masticatory Muscles and Jaw Function*, and *Oral Motor Function* (69% for each domain). The domains that were most-frequently reported in subjects with SMA type 3 were *Facial Expression* (50%), and *Habits, Chewing and Swallowing, Masticatory Muscles*, and *Jaw Function* (38% for each domain).

### NOT-S orofacial dysfunction profiles for myopathies

Diseases within the group of myopathies with more than 10 registered individuals were: DM1; Arthrogryposis Multiplex Congenita (AMC); Limb Girdle Muscular Dystrophy (LGMD); and DMD. Within these subgroups, LGMD demonstrated both the lowest proportion of individuals with signs of orofacial dysfunction (4/12, 33%) and the smallest variability in scores. In contrast, DM1 showed the highest proportion affected (35/40, 88%). In the AMC subgroup, 10/15 (67%) patients showed orofacial dysfunction. Forty-nine of the 69 (71%) subjects in the subgroup with DMD demonstrated orofacial dysfunction (Table 2).

**Figure 5.**
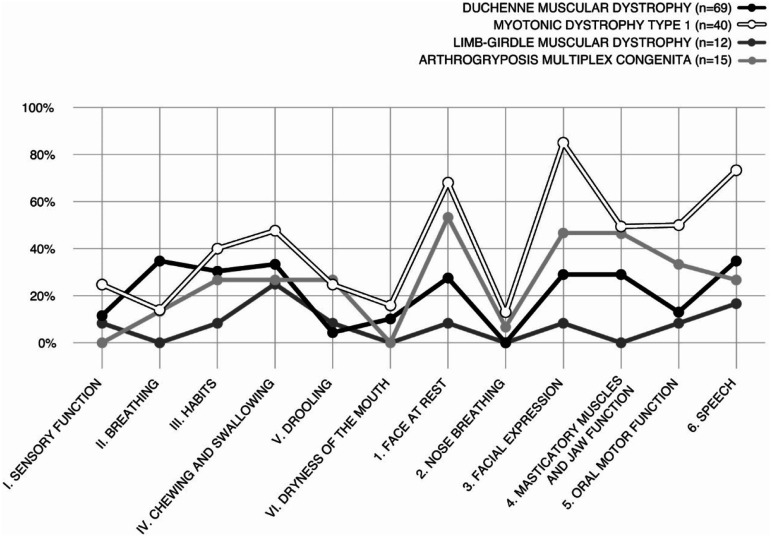
NOT-S orofacial dysfunction profiles with percentage of participants with dysfunction for each domain for the most common myopathies (including muscular dystrophies and arthrogryposis) in the registry sample from the MHC database.

In the LGMD subgroup, dysfunctions were most commonly observed in the domains of *Chewing and Swallowing* (28%), *Speech* (17%) and *Sensory Function, Habits, Drooling, Face at Rest, Facial Expression and Oral Motor Function* (8%). In the DM1 subgroup, the most-common dysfunctions occurred in the domains of *Facial expression* (83%), *Speech* (73%), and *Face at Rest* (68%). In the AMC subgroup, the most-common dysfunctions were in the domains of *Face at rest* (53%), and *Masticatory Muscles and Jaw Function* and *Facial Expression* (47% for each domain). In the DMD subgroup, *Breathing, Speech* (35%) and *Chewing and Swallowing* (33%) were the domains in which the largest proportion exhibited dysfunctions (Figure 5).

### NOT-S orofacial dysfunction profiles of patients with myotonic dystrophy type 1

**Figure 6.**
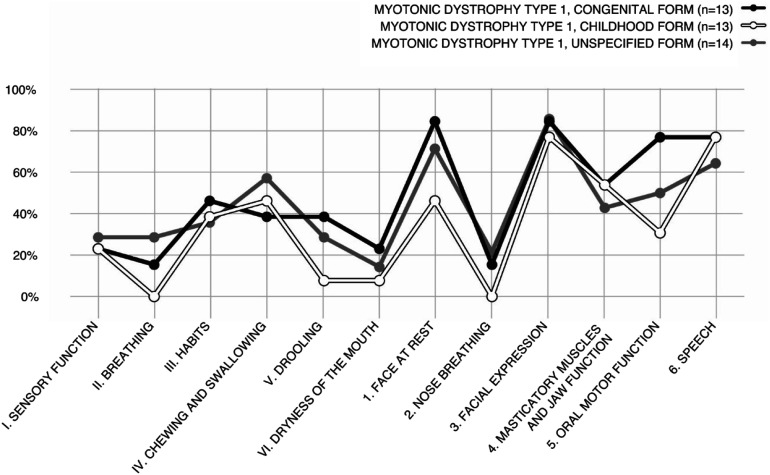
NOT-S orofacial dysfunction profiles with percentages of participants with dysfunction for each domain of myotonic dystrophy type 1, divided into disease forms. Data extracted from the MHC database.

Since DM1 comprises several different disease forms, the data were analyzed based on cases with a known disease form at the time of the examination. In the subgroup with the DM1 congenital form, 11/13 (85%) exhibited orofacial dysfunction. In the subgroup with the childhood form of DM1, 10/13 (77%) had orofacial dysfunction. Among those with the unknown form of DM1 (DM1 unspecified subgroup) 12/14 (86%) had a NOT-S result that signified orofacial dysfunction (Figure 6).

In the DM1 congenital form subgroup, the most-common dysfunctions occurred in the domains of *Face at Rest*, and *Facial Expression* (85%), and *Speech* (77%). In the childhood form of DM1, the most-common dysfunctions were in the domains of *Facial Expression*, *Speech* (77%) and *Masticatory Muscles and Jaw Function* (54%). Among those with the unknown form of DM1, dysfunctions in the domains of *Facial Expression* (86%), and *Face at Rest* (71%), and *Speech* (64%) were those with the highest prevalence.

### NOT-S orofacial dysfunction profiles of patients with Duchenne Muscular Dystrophy

Due to the progressive nature of the disease, the results for patients with DMD were also reported for different age groups. In DMD age group 3–10 years, 19/28 (68%) presented with orofacial dysfunction. For patients with DMD in the age group of 11–18 years, 22/33 (67%) had NOT-S values that signified orofacial dysfunction. Among those aged 19-49 years, the prevalence of scores indicating orofacial dysfunction was 8/8 (100%)

**Figure 7.**
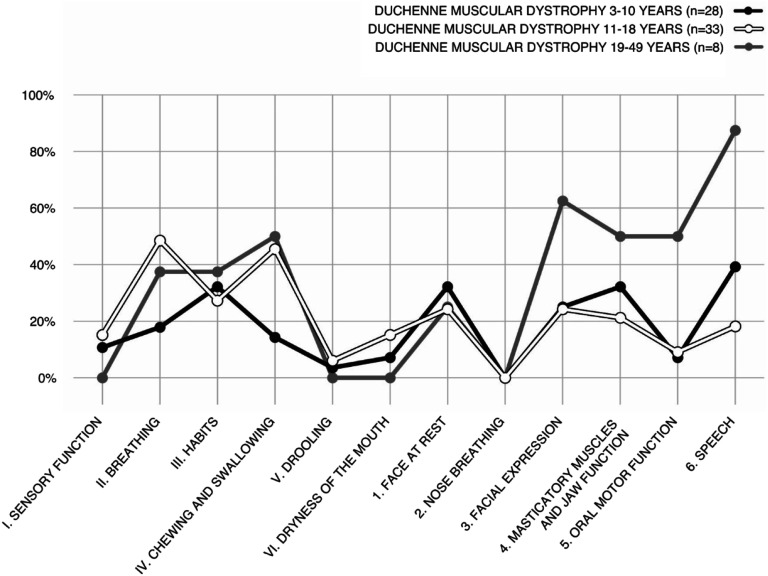
NOT-S orofacial dysfunction profiles with percentage of participants with dysfunction for each domain for Duchenne Muscular Dystrophy divided into age groups. Data extracted from the MHC database.

The most common dysfunctions in the age group of 3–10 years were in the domains of *Speech* (39%), and *Habits, Face at Rest, Masticatory Muscles and Jaw Function* (32%). Dysfunctions in the following domains were most common in the age group of 11–18 years: *Breathing* (48%), *Chewing and Swallowing* (45%), and *Face at Rest* and *Facial Expression* (24%). Among those patients with DMD aged 19–49 years, the most commonly reported dysfunctions occurred in the domains of: *Speech* (88%), *Facial Expression* (63%), and *Chewing and Swallowing*, *Masticatory Muscles and Jaw Function* and *Oral Motor Function* (50%) (Figure 7).

## Discussion

The aim of this study was to explore orofacial functions from a broad perspective in individuals with congenital or childhood-onset NMD, and to identify the proportion of those showing signs of orofacial dysfunction as well as differences between groups and subgroups. In some of the diagnoses included in the study, such as FSHD, DM1, SMA type 1, and nemaline myopathy, impact on various orofacial functions is well known,^9,16,23,33^ while in other groups like neuropathies and AMC orofacial functions are not as well explored.^13^ Neuropathies showed the lowest proportion among the groups studied, yet signs of orofacial dysfunction were still observed in 3/12 (25%). In AMC, 10/15 (67%) showed a result corresponding to orofacial dysfunction. The greatest difference in the occurrence of signs of orofacial dysfunction was found between Neuropathies and Anterior Horn Cell diseases, SMA type 1, i.e., diseases that primarily affect distally via the long nerve pathways, and the bulbar impact in SMA 1 with pronounced weakness in the neck, throat and respiratory muscles which in its natural course is among the first symptoms.^6^ However, the main findings of this study were that there is a high prevalence (70%) of NOT-S total dysfunction scores equivalent with orofacial dysfunction in the total study population with congenital or childhood-onset NMD. The most common orofacial difficulties for the total study population occurred in the domains *Facial expression, Speech, Face at rest,* and *Chewing and Swallowing*. Across all groups there were both individuals who presented with as well as without orofacial difficulties, except for in the groups SMA 1 and DMD 19-49 yrs., where all participants (100%) presented with scores indicating orofacial dysfunction.

For the total study population, the results for the domains exploring symptoms of dysphagia (*Chewing and Swallowing* and *Masticatory Muscles and Jaw Function*) are slightly lower than previous research on dysphagia prevalence in NMD as a group.^12^ This study also found a slightly higher rate of speech difficulties than previously reported.^12^ It is difficult to compare the results of this study with previously published research as differences may be due to results from individuals with different NMD or how the functions are examined. The fact that the occurrence differs from previous studies that sought to investigate the prevalence of, for example, dysarthria and dysphagia is therefore not surprising. Similarly, the definition of dysphagia, dysarthria, and bulbar function varies between studies, but as described in the introduction, orofacial function is a broad concept covering functional difficulties as well as the consequences of these problems at the activity level. Whether orofacial functions are affected or not is mostly dependent on the degree of oral motor/bulbar involvement, but respiratory function, general health, structural abnormalities and neurodevelopmental delay also influence these functions.

Previously, it has been reported that there is a lack of studies on anterior horn cell disease,^13^ but in recent years, articles on SMA have increased significantly in line with new drug treatments. In the anterior horn cell disease group (SMA types 1-3), SMA type 1 presented with the most-severe orofacial dysfunctions, whereby all the participants had a NOT-S dysfunction score ≥2. SMA type 2 showed a similar pattern, in that 92% presented with signs of orofacial dysfunction. SMA types 1 and 2 followed each other in their respective orofacial dysfunction profiles, while SMA type 3 exhibited a different profile, with the most common difficulties occurring in the domain of facial expressions. This is somewhat surprising, as several previous studies comparing SMA types 2 and 3 have found similar difficulties of varying severity,^8,17^ albeit with faster progression of symptoms in SMA type 2.^34^ The impact on several orofacial functions in the natural course of SMA is well known.^6,34^ In this study, this was the case even though six out of seven participants with SMA type 1 and six out of thirteen with SMA type 2 received disease-modifying treatment, with Nusinersen or Risdiplam. Previous studies have shown that children with SMA type 1 who underwent treatment experienced improved general motor development, although orofacial function did not seem to improve to the same extent.^35,36^ Early treatment, before the onset of symptoms, is of the greatest importance in terms of preserving motor functions.^37^ Information on the timing of treatment initiation for the individuals with SMA involved in the present study is lacking. This would have been of great interest, as it likely would affect the results and provide more-accurate information on the orofacial functions of individuals with SMA. There are studies showing that when gene therapy is administered before the onset of symptoms, normal bulbar function is achieved.^38^ None of the children in the present cohort had received gene therapy. Given the importance of early identification and treatment, SMA has been since 2023 included in the Swedish newborn screening program.^39^ This screening program was not in place during the period when individuals with SMA included in this study were born. Perhaps in the future, neonatal screening will entail that some children with SMA will achieve normal bulbar function, without orofacial dysfunction, while others, in whom the disease processes have already started, perhaps even during fetal life, will show orofacial dysfunctions. Therefore, children who are identified with SMA and who are medicated according to current guidelines need to be investigated further in relation to orofacial functions.

In this study, individuals with neuropathies were on a group level overall significantly less affected than individuals with anterior horn cell diseases and myopathies. There are very few studies on orofacial function in neuropathies.^13^ Kooi et al.^12^ reported a prevalence of dysphagia (23%) and dysarthria (18%) in twenty-four individuals with neuropathies, mainly individuals with Hereditary Motor Sensory Neuropathy. In the present study, neuropathies had the lowest occurrence of orofacial dysfunction according to NOT-S (3/12, 25%), 8% in the domains measuring dysphagia and 15% for domain 6, *Speech*. A possible explanation for this difference is that the subjects with neuropathy in this cohort the most-common diagnosis is Charcot-Marie-Tooth disease. The course of Charcot-Marie-Tooth disease is, in most cases, slowly progressive and is characterized by gradual muscle atrophy and loss of sensation mainly in the distal muscles affecting the feet, lower legs, hands, and forearms.^40^ That the neuropathy subgroup stands out as being less affected may be because it comprises children who are most likely early in the course of the disease, such that atrophy has not yet developed in the muscles crucial for speech and eating, such as the respiratory muscles. However, the symptoms that were most common in the subjects with neuropathies were low facial muscle tone and impaired facial expressions. *Facial Expression* was affected in a large proportion of the subjects in several subgroups of the study population. For specific diagnoses, such as FSHD and DM1, this is known phenomenon,^9,41^ although it is less well understood for other diagnoses. Facial expressions are important for communication, for showing emotions such as joy and sadness, and for social participation.^9,11^ In future studies, it would be valuable to investigate how difficulties with mimicry affect individuals with different NMD and how these functions can be treated to optimize the activities, social participation, and quality of life of individuals.

It is common for speech and voice to be negatively affected in several different NMD.^7,9,15,23,42,43^ A weak voice, weak, limited and imprecise articulation, flattened prosody, and hypernasality are all factors that may contribute to reduced intelligibility. Speech difficulties can be due to orofacial impairments causing weakness of the muscles involved in speech, and also due to the requirement for ventilatory support. In the NOT-S, because it is a screening tool that only indicates whether signs of difficulties are present, the results for the *Speech* domain cannot discriminate between different types of speech difficulties, such as dysarthria, dyspraxia, and voice disorders. However, the results indicates that speech impairments are very common in NMD. It is likely that the type of speech difficulty varies across the different diagnoses and the disease subgroups. Similarly, there may be language difficulties, particularly in groups in which cognition and behaviors are often affected, such as DM1 and DMD.^44,45^ There is also increasing literature regarding emerging cognitive phenotypes in the treated SMA population.^46^ In this material, language is not distinguished from speech disorders. The results raise new questions about speech, voice, and language functions in patients with NMD and these topics need to be investigated in future studies.

Difficulties in domain *Face at rest* contains items Asymmetry, Deviant lip and tongue position and Involuntary movements and are some of the most common findings in the material. Having an open mouth at rest, that is mouth breathing, can in turn cause both dry mouth and negative development of the bite and affect masticatory function.^29^ For the childhood and congenital forms of DM1, an open mouth at rest and weak, hypotonic orofacial muscles are characteristic traits. Also, in FSHD and nemaline myopathy weak facial muscles is a well-known symptom,^9,33^ which is confirmed in the present study. However, why this is the case in other diseases such as neuropathies and DMD, is not known. In more advanced DMD, enlargement of the tongue is seen, which could lead to the need to keep the mouth open to accommodate the tongue.^27^ Reduced neck stability can cause the head to tilt backward, stretching the throat and opening the mouth. If these two theories could explain the symptoms, then open mouth at rest would be more common with increasing age, but in this material, open mouth at rest is most common in the group of youngest children with DMD. The same applies to Charcot-Marie-Tooth disease, which accounts for the majority of the neuropathy group, where the individuals in the study are between four and 15 years old. The question therefore remains. Young children are more prone to having colds and may have enlarged tonsils, causing them to breathe through their mouths. Tonsils are not assessed in NOT-S. The awareness of being assessed, as in the situation of NOT-S examination, is likely to increase with age. Fatigue is a symptom in several NMD, and muscle weakness can cause tiredness leading to have an open mouth posture. Similarly, respiratory problems are a common symptoms, and shortness of breath may require breathing through the mouth.

This study confirms that signs of dysphagia is common in cases of NMD, and that chewing is negatively affected in a large proportion of individuals in the total NMD group and for the majority of the different diseases included in the material.^5,12,13^. Chewing ability depends not only on oral motor and sensory function, but also on the status of the bite and occlusion. Malocclusion is common in several NMD.^27,29^ It is known that the orofacial anatomy and orofacial functions interact and influence each other.^17,47^ Therefore, the orthodontist is an important team member in NMD care.

The NOT-S domain *Masticatory Muscles and Jaw Function* consists of two items, assessing the active muscle tone and symmetry of the M. masseter and measuring maximum mouth opening. All of the groups studied, with the exception of the LGMD group, showed scores that indicated either affected masticatory muscles and/or impaired jaw function. A reduced mouth opening can negatively affect several important functions, such as chewing, speech, teeth brushing, and dental care, and it can also be a complication when there is a need for anesthesia.^19,34,48^ However, studies have shown that impaired jaw mobility in some cases of NMD can be successfully prevented by regular stretching.^34^ This result indicates that individuals with congenital or early-onset NMD should be examined regarding dysfunctions of the masticatory musculature and jaw.

In AMC, for all the types of DM1 and the DMD age group of 19–49 years, the interview section of NOT-S revealed lower proportions with impacts on the domain of *Chewing and Swallowing*, while the examinations showed higher proportions of dysfunctions in the domain of *Masticatory Muscles and Jaw Function*. This finding can be interpreted as being in line with previous research showing that in many cases, caregivers are unaware of the symptoms of orofacial dysfunction and eating difficulties^12,17,20^ and that individuals with NMS are likely to make gradual adjustments in relation to their abilities and therefore may not think that they have difficulty chewing.

Although not common, some individuals showed difficulties in the *Sensory Function* domain. Apart from certain neuropathies, these diagnoses are not expected to primary affect sensory function, and the domain may reflect defensive behavior rather than true sensory impairment, which is more typical of neuropsychiatric conditions such as autism spectrum disorder. The reason for this theory is because the highest occurrence is found in the DM1 and DMD groups, where previous studies show that autism and ADHD are common.^44,45^ Interestingly, these occurrences correspond with impaired saliva control in these groups, but this correlation does not apply to AMC and SMA 1, where no one reports impairment in sensory function, but 27% respectively 43% report problems with drooling. This may indicate that impaired saliva control can have different causes in different NMDs. In previous studies, a need for more knowledge about impaired saliva control in NMD has been articulated.^18^ In the present study population, no major problems with reduced saliva control were detected, except in the congenital DM1 (38%), AMC (27%), and SMA 1 (43%) groups. In other groups with more severely impaired motor functioning, such as cerebral palsy, difficulties with saliva control often occur in combination with swallowing difficulties.^49^ Many of the participants in the present study had extensive motor impairments and swallowing difficulties. This raises the question as to whether there are protective factors, respectively risk factors for reduced saliva control in segments of the NMD population. While cognitive functions, sensory functions, muscle tone and the way the tongue is affected by the disease probably play important roles in this, to investigate this issue, in-depth studies of each factor are needed. Although impaired saliva control appears to be much more common in a limited cohort of NMD, such as DM1, AMC and SMA1, these difficulties are important to address and take seriously for those affected, as they can have significant negative impacts on the individual and, for many cases, there are several available treatment options.^50,51^

In the self-assessed domain of *Dryness of the mouth* (xerostomia), a very low percentage of the participants reported dysfunction. This despite the fact that, in the present study, an open mouth at rest and chewing difficulties were frequently found, factors that in other studies have been shown to cause dry mouth.^52,53^ A possible explanation for this is that the respondents have a chronic disease and, therefore, may not know the difference between a dry mouth and the normal status, as this is that they have always experienced. Saliva secretion can be measured instrumentally. It would be of interest to investigate this finding further, given that reduced quantity and quality of the saliva can negatively affect speech, oral processing and swallowing.^52,53^ Under the same domain, there is the question “Do you have to drink to be able to eat a cracker?”. A positive outcome on this item among individuals with NMD could indicate signs of swallowing difficulties in the pharyngeal phase and pharyngeal residue, for example due to insufficient relaxation of the upper esophageal sphincter^18^ rather than lack of saliva. However, the prevalence of difficulties in domain *Dryness of the mouth* is lower in all groups and subgroups than the prevalence of reported outcomes in domain *Chewing and Swallowing.*

While there are several tests that can be used to examine oral motor skills, speech, eating or saliva control separately, few protocols provide a broader picture of orofacial function. This is the case despite the fact that various impairments of orofacial function tend to co-occur.^4,5,47^ Recently, a screening tool for dysphagia and/or dysarthria, for children and young adults with NMD, was developed to identify those who should be referred to an SLP.^21^ There is ongoing work to develop test materials for eating difficulties in young children with SMA 1,^25^ to investigate and monitor dysphagia^24,54^ and dysarthria^12^ in cases of NMD. However, in-depth tests to examine a broader range of orofacial functions for different NMDs and ages are, to the best of the authors’ knowledge, lacking. Despite its general applicability as a screening instrument, the NOT-S seems to capture differences between diagnoses, disease forms and age groups. Thus, the NOT-S can be a relevant screening tool for orofacial dysfunctions in NMD.

Based on the results of this study, several knowledge gaps have been identified. There is a need to establish which, and to what extents, orofacial problems are caused by a primary oral motor impairment, and which are of a more secondary nature, such as axial/neck weakness, respiratory weakness or reduced mobility in another part of the body. This knowledge is needed to be able to provide adequate treatment. There is a lack of understanding as to how orofacial dysfunctions develop over time in the various congenital or childhood-onset NMD. Furthermore, an in-depth analysis of communication difficulties in NMD is needed, both in terms of the prevalence rates and types of difficulties. Further research should include the patient’s perspective and explore how orofacial dysfunction affects the individual’s ability to be active and participate in daily life.

Over the last few years, there have been major changes in medical treatments, with the emergence of disease-modifying therapies for several NMD.^35,37,38,55–57^ These medical improvements require continuous scientific and clinical follow-ups of orofacial dysfunction. An in-depth standardized test material for the whole spectrum of orofacial functions is needed to monitor orofacial dysfunctions in congenital or childhood-onset NMD.

### Limitations

A shortcoming of this study is the different sizes of the groups. However, as the study concerns rare health conditions, it is important to note that the sizes of the groups in relation to the total prevalence of their diseases constitute an extensive material.

The present dataset did not contain information about AMC subtypes, which can vary in etiology and may be secondary to other underlying conditions. We chose to include AMC because clinical experience tells us that many people with AMC have orofacial dysfunctions (e.g., trismus) and because a previous literature review found no reports on orofacial function in individuals with AMC.^13^ The results of this study confirm this clinical experience but also show that the median NOT-S value for the group with AMC was relatively low and that the variability within the group was broad. For this reason, in future studies, it would be interesting to distinguish subgroups of AMC from each other. The MHC database contains no information about disease-causing genetic variation, which would have been useful in drawing more reliable conclusions.

The inter-rater reliability for the *Speech* domain is considered low, and the results for this domain should be interpreted with caution. Previous research has demonstrated that the reliability of perceptual assessments of speech can be low.^58^ This is especially true for nasality, and nasality is included in the domain of *Speech* in the NOT-S. It has been reported that listeners tend to create their own internal standard for assessment, and it has also been shown that when there are zero or minor problems, it is easier to make consistent judgements.^59^

### Clinical implications

Signs of orofacial dysfunction are frequently observed in both children and adults with congenital and childhood-onset NMD, as shown in this study. Patients with different forms of NMD have disease-specific orofacial dysfunction profiles. Since orofacial dysfunction can have a major negative impact on an individual’s wellbeing and participation in social activities, it needs to be addressed in NMD care. In some diseases, such as DMD, BMD, LGMD and certain neuropathies, orofacial function can be relatively well preserved in relation to muscle weakness in other parts of the body,^1^ healthcare providers should though be aware of possible orofacial dysfunction that requires further investigation. Expert knowledge and thorough clinical examinations of the anatomy and physiology of the mouth and face, as well as of treatments for orofacial dysfunctions are prerequisite skills for NMD teams. In particular, SLP expertise,^60^ as well as ready access to dental care are recommended. NOT-S is a relevant screening tool for orofacial dysfunction in cases of NMD.

### Conclusions

This study indicates a high occurrence of orofacial dysfunction in congenital or childhood-onset NMD, among both children and adults. Different NMD and their subgroups show diverse frequencies of dysfunctions and specific orofacial dysfunction profiles. Neuropathies are associated with the fewest orofacial dysfunctions, while anterior horn cell diseases show the highest prevalence of signs of orofacial dysfunctions according to NOT-S. However, in all the groups studied, with the exceptions of the SMA 1 and DMD 19–49 years old subgroups, there were individuals who did not report any self-perceived difficulties or who had no findings of orofacial dysfunction during the clinical examination.

## Supplemental material

Supplemental material - Orofacial dysfunction in persons with congenital or childhood-onset neuromuscular disorders Supplemental material for Orofacial dysfunction in persons with congenital or childhood-onset neuromuscular disorders by Lisa Bengtsson-Stelzer, Anna-Karin Kroksmark, Christina Persson, Lisa Tuomi, Anne-Berit Ekström in Journal of Neuromuscular Diseases.

## Data Availability

The authors confirm that the data supporting the findings of this study are available within the article. The data will be shared on reasonable request to the corresponding author.*
