# Supplementary material for: Orofacial dysfunction in persons with congenital or childhood-onset neuromuscular disorders
Source: J Neuromuscul Dis. 2026 May 16:22143602261452338. Online ahead of print. doi: 10.1177/22143602261452338 (PMC13437852; doi:10.1177/22143602261452338)
Supplement: Supplementary file 1 [file sj-pdf-1-jnd-10.1177_22143602261452338.pdf]

Supplementary I.

Domains assessed in the Nordic Orofacial Test–Screening (NOT-S). The structured interview is conducted via questionnaire or verbally. The tasks in the examination domains are given verbally and are supported by pictures. The scoring for each item is based on "yes" or "no" answers. One or more positive answers within a domain generates one dysfunction score. The maximum NOT-S dysfunction score is 12. NOT-S is free of charge and available for download (32).

NOT-S interview domains I-VI	*NOT-S examination domains 1-6*
I. Sensory function	*1. Face at Rest*
A: Does brushing your teeth elicit a gag reflex (every time)?<br>B: Do you put so much food into your mouth that it becomes difficult to chew (every day)?	A: Asymmetry<br>B: Deviant lip position<br>C: Deviant tongue position<br>D: Involuntary movements
II. Breathing	*2. Nose Breathing*
A: Do you use any breathing support?<br>B: Do you snore much when you sleep (every night)?	A: Close your mouth and take five deep breaths through your nose
III. Habits	*3. Facial Expression*
A: Do you bite your nails or suck your fingers or other subjects every day?<br>B: Do you suck or bite your lips, your tongue or your cheeks every day?<br>C: Do you bite your teeth together hard or grind your teeth during the day?	A: Close your eyes tightly (symmetrically)<br>B: Show your teeth (symmetrically, teeth visible)<br>C: Try to whistle (cannot pout and round the lips)
IV. Chewing and Swallowing	*4. Masticatory Muscles and Jaw Function*
A: Does not eat with the mouth (artificial nutrition via nasogastric tube, g-tube, jejunostomy, or total parenteral nutrition)<br>B: Do you find it difficult to eat foods with certain consistencies?<br>C: Does it take you 30 min or more to eat a main meal?<br>D: Do you swallow large bites without chewing?<br>E: Do you often cough during meals?	A: Bite hard on your back teeth<br>B: Open your mouth as wide as you can

V. Drooling	*5. Oral Motor Function*
A: Do you get saliva in the corner of the mouth or on the chin almost every day?	A: Stick out your tongue as far as you can<br>B: Lick your lips<br>C: ‘Blow up” your cheeks and hold for at least 3 s<br>D: Open your mouth wide and say ah-ah-ah (elevation of the uvula)
VI. Dryness of the Mouth	*6. Speech*
A: Do you have to drink to be able to eat a cracker?<br>B: Do you suffer from pain in the mucous membrane in your mouth or on your tongue (not blisters)?	A: Does not speak<br>B: Count out loud to 10 (unclear articulation, hypo- or hypernasality, deviate resonance) *<br>C: Say pa-ta-ka, pa-ta-ka, pa-ta-ka*

*In children aged between 3 and 5 years, the speech assessment is adjusted for age.

Supplementary II.

NOT-S results presented with the percentages of cases with dysfunction for each domain in the groups of congenital or childhood-onset neuromuscular diseases.

	NOT-S interview I-VI						*NOT-S examination 1-6*					
	I. Sensory function	*II. Breathing*	*III. Habits*	*IV. Chewing and Swallowing*	*V. Drooling*	*VI. Dryness of the Mouth*	*1. Face at Rest*	*2. Nose Breathing*	*3. Facial Expression*	*4. Masticatory Muscles and Jaw Function*	*5. Oral Motor Function*	*6. Speech*
NMD as one group	12%	27%	28%	41%	15%	8%	**44%**	7%	**45%**	38%	30%	**44%**
[a]Anterior horn cell dis	4%	43%	18%	**68%**	14%	4%	**61%**	7%	54%	**64%**	54%	57%
[c]SMA1	0%	**86%**	14%	**100%**	43%	0%	**86%**	14%	**71%**	**86%**	**86%**	**86%**
[c]SMA2	8%	38%	8%	**69%**	0%	8%	**69%**	0%	46%	**69%**	**69%**	62%
[c]SMA3	0%	13%	**38%**	**38%**	13%	0%	25%	13%	**50%**	**38%**	0%	25%
[a]Neuropathies	0%	8%	0%	8%	0%	0%	**23%**	15%	**23%**	8%	8%	**15%**
[a]Myopathies	14%	25%	31%	39%	16%	9%	**43%**	6%	**45%**	36%	28%	**44%**
[b]LGMD	8%	0%	8%	**28%**	8%	0%	8%	0%	8%	0%	8%	**17%**
[b]AMC	0%	13%	27%	27%	27%	0%	**53%**	7%	**47%**	**47%**	33%	27%
[b]DM1	25%	15%	40%	48%	25%	15%	**68%**	13%	**83%**	50%	53%	**73%**
[c]DM1 congenital	23%	15%	46%	38%	38%	23%	**85%**	15%	**85%**	**54%**	**77%**	**77%**
[c]DM1 childhood	23%	0%	38%	46%	8%	8%	46%	0%	**77%**	**54%**	31%	**77%**
[c]DM1 unspec.	29%	29%	36%	57%	29%	14%	**71%**	21%	**86%**	43%	50%	**64%**
[b]DMD	12%	**35%**	30%	**33%**	4%	10%	28%	0%	29%	29%	13%	**35%**
[c]DMD 3–10 yrs.	11%	18%	**32%**	14%	4%	7%	**32%**	0%	25%	**32%**	7%	19%
[c]DMD 11–18 yrs.	15%	**48%**	27%	**45%**	6%	15%	**24%**	0%	**24%**	21%	9%	18%
[c]DMD 19–49 yrs.	0%	38%	38%	**50%**	0%	0%	25%	0%	**63%**	**50%**	**50%**	**88%**

[a] Groups based on primary disease location.
[b] Myopathies occurring in more than 10 individuals in the dataset.
[c] Subgroups of diseases divided according to disease forms or age groups.
Abbreviations: NMD, Neuromuscular diseases; SMA, Spinal Muscular Atrophy; LGMD, Limb-Girdle Muscular Dystrophy; AMC, Arthrogryposis Multiplex Congenita; DM1, Myotonic Dystrophy type 1; DMD, Duchenne Muscular Dystrophy (DMD).
Shading Key: Black, ≥75% showed difficulties in the domain; dark grey, ≥50% showed difficulties in the domain; Light grey, ≥25% showed difficulties in the domain. The numbers in bold represent the three highest values found for each group.
